# Skin Lesions Mimicking Mosquito Bites as the Initial Presentation of Chronic Lymphocytic Leukemia/Small Lymphocytic Lymphoma

**DOI:** 10.7759/cureus.112790

**Published:** 2026-07-16

**Authors:** Sami Alghayath, Noora Alali, Meera Alshirawi, Esmaeel AlMarzooqi

**Affiliations:** 1 Medicine, Mohammed Bin Rashid University of Medicine and Health Sciences, Dubai, ARE; 2 Dermatology, Rashid Hospital, Dubai, ARE; 3 Dermatology, Mohammed Bin Rashid University of Medicine and Health Sciences, Dubai, ARE; 4 Dermatology, Dubai Health, Dubai, ARE

**Keywords:** chronic lymphocytic leukemia (cll), dermatological manifestation, insect bite complications, mosquito bite, skin biopsy, small lymphocytic lymphoma (sll), misdiagnosis

## Abstract

We report the case of a 60-year-old man who initially presented in October 2021 with a history of recurrent itchy skin lesions across exposed areas of his body such as the neck and extremities. As per history and clinical examination, the lesions were initially interpreted as an insect bite reaction for which the patient was provided with symptomatic treatment. Despite the treatment, the patient continued to experience recurrent skin lesions. Histopathologic exam of a skin biopsy showed eosinophil-rich superficial and deep inflammatory infiltrates which supported the diagnosis of a cutaneous reaction to an insect bite or another hypersensitivity-type reaction which further made the diagnosis challenging.

In 2024, he developed systemic symptoms such as dizziness, vomiting, and generalized lymphadenopathy. Laboratory tests revealed significant leukocytosis with persistent lymphocytosis, mild anemia, and thrombocytopenia. A peripheral smear was done and showed mature lymphocytosis with smudge cells. Flow cytometry identified a clonal B-cell population expressing CD5, CD23, CD200, and lambda light-chain reaction, supporting a diagnosis of chronic lymphocytic leukemia. Positron emission tomography-computed tomography (PET-CT) demonstrated hypermetabolic lymphadenopathy above and below the diaphragm and hepatosplenomegaly.

Cutaneous manifestations resembling different benign dermatologic conditions, such as an insect bite reaction, may rarely precede systemic symptoms of an underlying hematologic malignancy. This case emphasizes the significance of reassessment and follow-up in patients with recurrent and evolving dermatological findings.

## Introduction

Chronic lymphocytic leukemia/small lymphocytic lymphoma (CLL/SLL) is a mature B-cell neoplasm with progressive accumulation of monoclonal B lymphocytes in the peripheral blood, bone marrow, and lymph nodes [1]. CLL is the most common form of leukemia in adults in Western populations, and patients may be asymptomatic at diagnosis [1]. Skin lesions associated with hematologic malignancies can present similarly to benign inflammatory skin lesions such as insect bite reactions and drug-related skin reactions which could potentially mask the true diagnosis, therefore delaying the treatment [2]. On rare occasions, patients may develop dermatologic manifestations due to immune dysregulation or leukemic infiltration [3].

There are several presentations of dermatologic manifestations associated with hematologic malignancies, including conditions known as urticarial eruptions, leukemia cutis, eosinophilic dermatosis, and papulovesicular lesions [2,3]. Eosinophilic dermatoses may clinically and histopathologically be similar to hypersensitivity reactions, such as skin reactions to insect bites, which would therefore mask the diagnosis of a hematologic malignancy and, in turn, delay the treatment [2].

We report the case of a patient who presented with recurrent itchy skin lesions across exposed areas of his body, initially interpreted as a hypersensitivity-type reaction clinically and histopathologically before eventual investigations revealed CLL/SLL several years later.

## Case presentation

A 60-year-old man initially presented to the emergency department in October 2021 with recurrent itchy, erythematous skin lesions involving the arms and neck which would exacerbate further in winter. The lesions involved localized swelling and irritation; however, it was not associated with difficulty breathing or lip swelling nor any lymphadenopathy. History and clinical assessment leaned towards a diagnosis of insect bite reaction, and the patient was provided with symptomatic treatment with oral antibiotics such as amoxicillin-clavulanate, topical antibiotics such as fusidic acid, oral steroids such as prednisolone, topical steroids such as hydrocortisone, and antihistamines such as levocetirizine.

Despite treatment, the lesions persisted and recurred; therefore, he was referred to the dermatology department where a thorough examination was done which revealed multiple erythematous papules and nodules of varying sizes involving the neck and the arms (Figures 1-3). Several lesions demonstrated central crusting and hyperpigmentation. He was then advised to do a skin biopsy for which histopathology later revealed eosinophil-filled superficial and deep inflammatory infiltrates composed of lymphohistiocytic cells which supported the diagnosis of a hypersensitivity-type reaction and was given symptomatic treatment.

**Figure 1:**
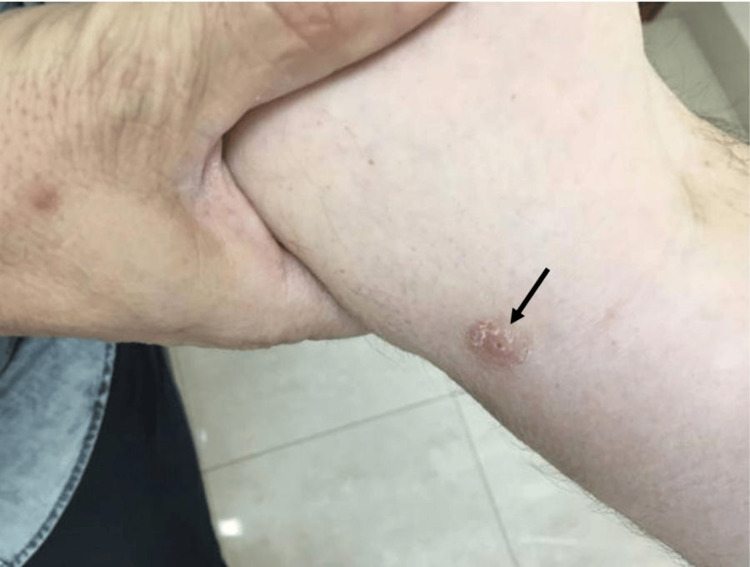
Erythematous papule with crusting on the forearm at the first dermatological encounter as shown by the black arrow

**Figure 2:**
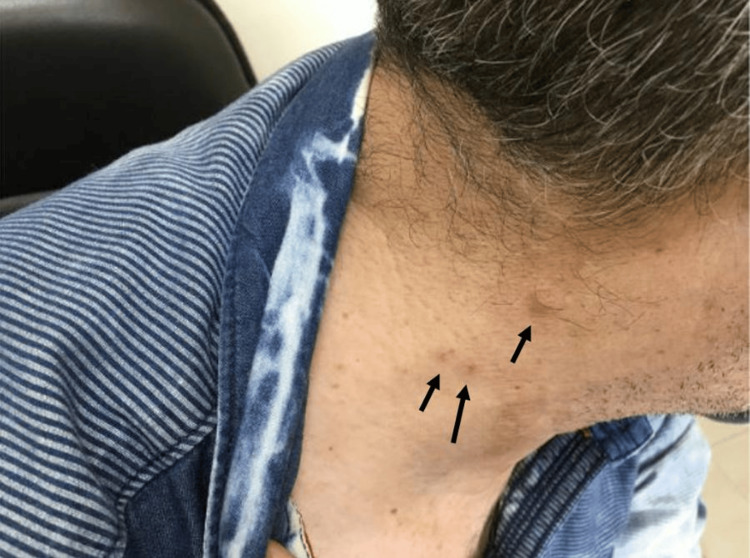
Multiple erythematous papules on the lateral neck at the first dermatological encounter as shown by the black arrows

**Figure 3:**
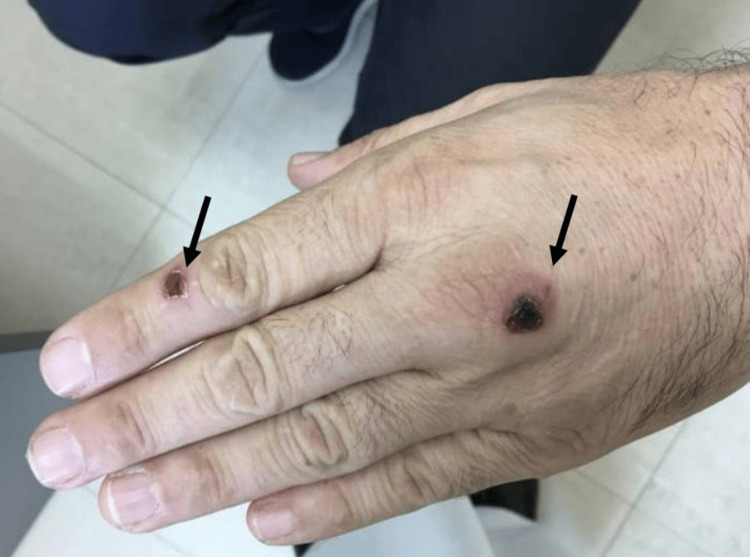
Crusted erythematous papules on the dorsal aspect of the hand and index finger at the first dermatological encounter as shown by the black arrows

The patient presented a week later to his first dermatologic visit, having developed bullous lesions on the neck and hand for which he was given oral antibiotics such as amoxicillin-clavulanate and stronger topical steroids such as clioquinol-betamethasone and was advised to have close follow-up in the clinic (Figures 4-5).

**Figure 4:**
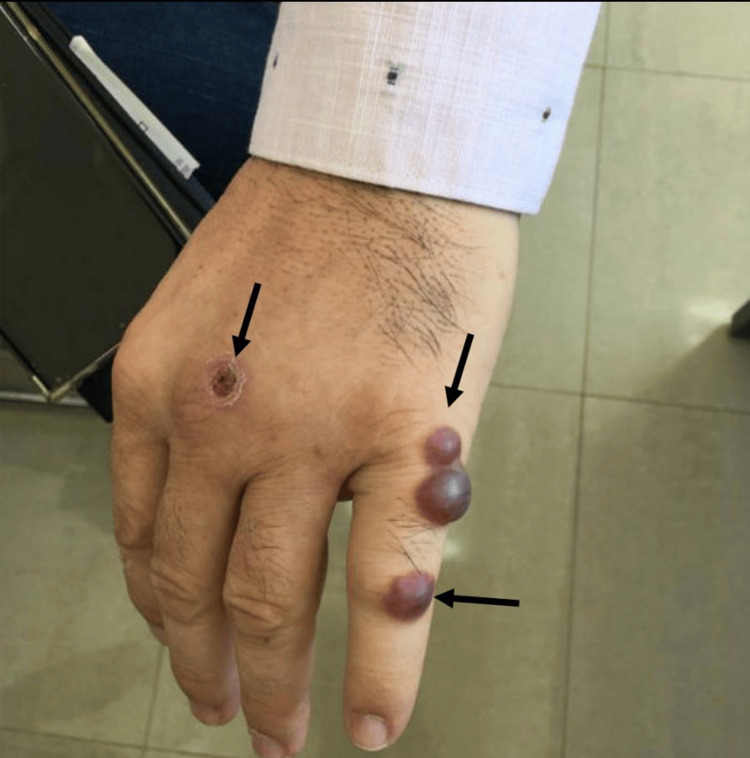
Bullous nodules on the pinky finger with a crusted papule on the dorsum of the hand which have worsened since the first dermatological presentation as shown by the black arrows

**Figure 5:**
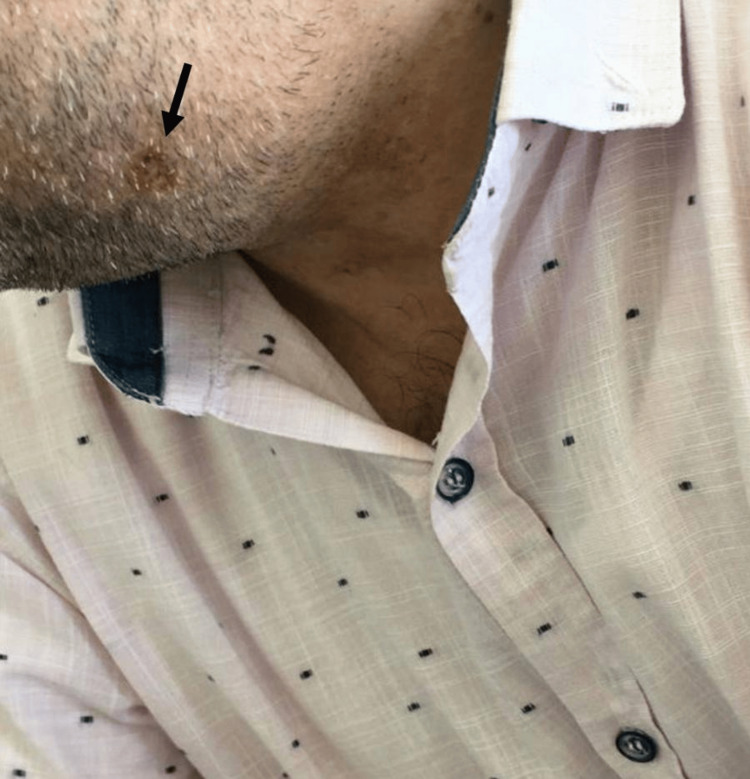
Erythematous papule with crusting on the left mandibular region as shown by the black arrow

In January 2026, the patient developed systemic symptoms, including dizziness, vomiting, and generalized lymphadenopathy, which in turn required further medical evaluation.

On questioning, he had visited a private clinic in 2024 for which a routine blood test had shown an elevated white blood cell count and was advised to do a workup in Syria, his home country, due to financial constraints. Workup in Syria revealed a white blood cell count of 82,000/µL, an absolute lymphocyte count of 71,000/µL, a hemoglobin of 13.7 g/dL, a mean corpuscular volume of 86.2 fL, a platelet count of 270,000/µL, flow cytometry revealing CLL, and a positron emission tomography-computed tomography (PET-CT) which showed lymphadenopathy above and below the diaphragm with hepatosplenomegaly.

A core needle biopsy of the right axillary lymph node was also done in April 2024, revealing monotonous atypical lymphoid cell proliferation, possibly relating to non-Hodgkin lymphoma. No Reed-Sternberg cells, granulomas, or necrosis were seen, however.

On his visit in January 2026, a CT imaging was advised and done which demonstrated lymphadenopathy involving the cervical, axillary, mediastinal, abdominal, pelvic, and inguinal regions associated with mild hepatosplenomegaly. A neck ultrasound was also done which demonstrated pathological cervical lymph nodes with increased vascularity and loss of fatty hilum.

A PET-CT was done again in January 2026 in the hospital which showed hypermetabolic lymphadenopathies above and below the diaphragm with mild hepatosplenomegaly and hypermetabolic activity in the L3 lumbar vertebral body, as identified before.

Laboratory evaluation in January 2026 demonstrated marked leukocytosis with white blood cell counts ranging from 75 to 93x10⁹/L associated with marked lymphocytosis, hemoglobin levels ranging between 10 and 11.5 g/dL with thrombocytopenia, and mildly elevated lactate dehydrogenase levels (Table 1).

**Table 1:** Summary of the documented hematologic investigations WBC: white blood cell count; LDH: lactate dehydrogenase test

Timeline	WBC (×10⁹/L)	Absolute lymphocyte count (×10⁹/L)	Lymphocyte (%)	Hemoglobin (g/dL)	Platelets (×10⁹/L)	LDH (U/L)
Reference range	4-11	1-3	20-40	13.5-17.5	150-450	140-280
Day 1 of admission to the internal medicine ward	82.2	68.23	83	10.3	128	299
Day 2	75.8	65.95	87	10.6	119	-
Day 3	83.9	-	-	10.1	125	-
Day 5	82.9	78.75	95	10.4	129	-
One-month follow-up visit with the hematology department	92.9	84.54	91	11.5	112	-

Peripheral blood smear in January 2026 revealed lymphocytosis with multiple smudge cells and prolymphocytes, leaning towards a diagnosis of a mature lymphoproliferative disorder.

Flow cytometry in January 2026 demonstrated a clonal B-cell population expressing CD19, CD20, CD5, CD23, CD200, and ROR1 with lambda light-chain restriction, without expression of CD10 and FMC7, further signifying diagnosis of CLL/SLL.

Based on the clinical picture and hematologic and radiologic findings, the patient was given a diagnosis of CLL/SLL and was advised to undergo regular follow-ups with the hematology department regarding his condition and possible treatment.

## Discussion

Skin manifestations that are associated with or precede CLL/SLL can vary and can also be caused by a variety of causes, such as immune dysregulation, eosinophilic dermatoses, leukemic infiltration, and paraneoplastic inflammations, or possibly even caused by the treatment itself [4-6]. Skin features may occasionally precede other systemic symptoms of hematological malignancies and therefore create a diagnostic challenge [4]. There have been reported cases where dermatologic features present after the diagnosis of CLL, however [5].

Eosinophilic dermatosis has been increasingly associated with CLL/SLL as well as other hematologic malignancies such as mantle cell lymphoma, large cell lymphoma, myelofibrosis, and acute monocytic leukemia [7]. Eosinophilic dermatoses are a group of diseases that present when there is an eosinophil-rich infiltrate and/or degranulation of eosinophils frequently without leukemic infiltration which would resemble a benign condition [8]. This condition typically presents with pruritic, papular, and sometimes vesicular lesions which present similarly to an insect bite, a drug reaction, or even a hypersensitivity reaction [7]. Different pathologies have been reviewed and suggested including altered immune regulation, malfunction in cytokine signaling, and overstated hypersensitivity reactions associated with the underlying lymphoid malignancy [9].

Previously reported cases have described patients presenting with recurrent bullae [10], exaggerated insect bites causing ulcers [11], papulovesicular lesions [6], and pruritic lesions [7]. Some of the cases preceded the diagnosis of the CLL similarly to our case [6,9], while the typical lesions appear months to years after the diagnosis of leukemia although still a rare presentation [9-11]. In cases where the skin manifestations happened prior to the hematologic diagnosis, it took months to years for the hematologic diagnosis to be made, and patients were falsely treated for long periods of time due to the initial masking of the disease and misdiagnosis.

In the case of our patient, his skin manifestations were further exacerbated during the winter season which additionally suggested a hypersensitive reaction and therefore prolonged the diagnosis further until accompanied by extensive lymphadenopathy and systemic symptoms at the time of diagnosis.

This case highlights the importance of having a broad diagnostic approach and maintaining a suspicion for underlying hematologic malignancies in patients who present with any dermatologic manifestation especially when they are recurrent and fail to respond to conventional treatment, as there are a variety of presentations as well as causes to such presentations.

Such cases need to be investigated through a multidisciplinary approach involving different departments early in case the disease is recurrent or does not respond to conventional therapy or if the diagnosis involves slight suspicion.

## Conclusions

Recurrent dermatologic lesions mimicking insect bite reactions or hypersensitivity reactions may occur prior to underlying hematologic malignancies. Recurrent, persistent, or resistant cases to treatment should prompt a suspicion regarding the initial diagnosis and, in turn, push physicians towards reassessment and consideration of other causes. This case highlights the different dermatological manifestations associated with CLL/SLL and emphasizes the critical analysis of suspicious cases through a multidisciplinary approach.
